# Long-term outcomes and prognosis of neuroendocrine neoplasms of the head and neck: a cohort from a single institution

**DOI:** 10.1007/s00432-024-05726-1

**Published:** 2024-06-04

**Authors:** Xinqi Shi, Xiaodong Huang, Kai Wang, Yuan Qu, Xuesong Chen, Runye Wu, Ye Zhang, Jianghu Zhang, Jingwei Luo, Jingbo Wang, Junlin Yi

**Affiliations:** 1 Department of Radiation Oncology, National Cancer Center/National Clinical Research Center for Cancer/Cancer Hospital, Chinese Academy of Medical Sciences and Peking Union Medical College, Beijing, China; 2 Department of Radiation Oncology, National Cancer Center/National Clinical Research Center for Cancer/Hebei Cancer Hospital, Chinese Academy of Medical Sciences (CAMS), Langfang, China

**Keywords:** Neuroendocrine neoplasm of the head and neck, Survival outcome, Treatment regimens, Radiotherapy

## Abstract

**Background:**

Neuroendocrine neoplasm is a rare cancer of head and neck. This study aimed to evaluate clinical features, treatment outcomes, and prognostic factors of neuroendocrine neoplasm of head and neck treated at a single institution.

**Methods:**

Between Nov 2000 and Nov 2021, ninety-three patients diagnosed with neuroendocrine neoplasms of head and neck treated at our institution were reviewed retrospectively. The initial treatments included chemotherapy (induction, adjuvant, or concurrent) combined with radiotherapy in 40 patients (C + RT group), surgery followed by post-operative RT in 34 (S + RT group), and surgery plus salvage therapy in 19 patients (S + Sa group).

**Results:**

The median follow-up time was 64.5 months. 5-year overall survival rate (OS), progression-free survival rate (PFS), loco-regional relapse-free survival free rate (LRRFS) and distant metastasis-free survival rate (DMFS) were 64.5%, 51.6%, 66.6%, and 62.1%, respectively. For stage I–II, the 5-year LRRFS for patients’ treatment regimen with or without radiotherapy (C + RT and S + RT groups versus S + Sa group) was 75.0% versus 12.7% (*p* = 0.015) while for stage III–IV, the 5-year LRRFS was 77.8% versus 50.0% (*p* = 0.006). The 5-year DMFS values for patients with or without systemic therapy (C + RT group versus S + RT or S + Sa) were 71.2% and 51.5% (*p* = 0.075). 44 patients (47.3%) experienced treatment failure and distant metastasis was the main failure pattern.

**Conclusions:**

Radiotherapy improved local–regional control and played an important role in the management of HNNENs. The optimal treatment regimen for HNNENs remains the combination of local and systemic treatments.

## Introduction

Neuroendocrine neoplasm is a rare and heterogeneous tumor type (Rindi et al. 2022), comprising about 2% of all malignancies, with the most common site of lung (Oronsky et al. 2017; Rekhtman 2022). Primary neuroendocrine neoplasms of the head and neck (HNNENs) are rare, accounting for only 0.3% of all head and neck cancers (Ohmoto et al. 2021; Rindi et al. 2022). Due to the low incidence, studies describing features and management of HNNEN remain limited.

The World Health Organization (WHO) 2022 Classification of head and neck tumors divides HNNENs into well-differentiated neuroendocrine tumors of head and neck (HNNETs) and poor-differentiated neuroendocrine carcinomas of the head and neck (HNNECs) (Mete and Wenig 2022). HNNETs are generally graded as G1, G2, and G3 based on proliferation, and HNNECs are sub-classified as small or large cell types according to cell morphology (Mete and Wenig 2022). Moreover, the widely accepted classification, which divided HNNETs into typical carcinoids and atypical carcinoids, is previously used.

Treatment modalities, including surgery, radiotherapy, chemotherapy, or the combination of various treatments, have been used for patients with different conditions (Matsuyama et al. 2022; Mitchell et al. 2021; Strojan et al. 2019). However, there is no consensus on the optimal treatment strategy for HNNENs. Since the occurrence of HNNENs is sporadic, it is hard to perform large clinical trials. Thus, we performed this retrospective study with large sample size, aiming to assess the long-term outcomes and prognosis and further explore the role of radiotherapy in the treatment of HNNENs.

## Methods

### Patient data

A retrospective review of all patients with diagnoses of HNNENs at our institution between November 2000 and November 2021 was performed. This study was approved by the local ethics committee. A total of 93 patients with pathologically proven HNNENs were included in this study. All diagnoses were confirmed by two independent pathologists in our hospital. The TNM stage classification was evaluated according to the Staging Manual of American Joint Committee on Cancer (AJCC) or Union for International Cancer Control (UICC) for different location and determined by clinical and imaging examinations, including CT or MRI.

### Treatment

Among 93 patients, 40 patients underwent chemotherapy and radical radiotherapy (C + RT group: 33 of them underwent induction chemotherapy, all 40 had concurrent chemotherapy, 10 of them received adjuvant chemotherapy), 34 patients had radical surgery combined with post-operative radiotherapy (S + RT group: 14 of them also received concurrent chemotherapy) and 19 patients underwent primary surgery alone followed with salvage treatment (S + Sa group).

All patient treatment strategies were recommended by our multidisciplinary board. The recommended treatment for early disease was surgery alone or concurrent chemo-radiotherapy. For those at high risk of recurrence after surgery, including those with positive or close surgical margins, or pathologically positive lymph node, post-operative RT was suggested and delivered at 4–6 weeks after the surgery. As for those with unresectable disease or large tumor burden by imaging examinations, induction chemotherapy followed with radical radiotherapy was recommended. The radical radiation dose of gross tumor volume (GTV) was about 70 Gy, and the clinical target volume (CTV) about 60 Gy. The median CTV dose of post-operative RT was 60.06 Gy, ranging from 45 to 64 Gy. The regimen for chemotherapy was mostly cisplatin-based combination regimen, typically etoposide plus cisplatin (EP).

### Statistical analyses

All events (including death, progression, loco-regional failure, and distant metastasis) were measured from the date of diagnosis until clearly documented first-time failure, corresponding to overall survival rate (OS), progression-free survival rate (PFS), loco-regional relapse-free survival free rate (LRRFS), and distant metastasis-free survival rate (DMFS). Categorical data were compared using the chi-square test. The survival data were estimated using Kaplan–Meier method. All survivals were compared using the log-rank test. Multivariate analysis using the Cox proportional hazard model was performed to identify the prognostic factors. All reported *p* values were two-sided, and *p* values below 0.05 were considered to be significant.

## Results

### Patient characteristics

Among the 93 patients, there were 71 male and 22 female, with a median age of 53 years (range from 18 to 82 years). The most common primary sites were as follows: nasal cavity (25 patients), larynx (23 patients), paranasal sinus (19 patients), and nasopharynx (9 patients). The other sites included oropharynx, oral cavity, saliva gland, thyroid, and unknown original site. According to T stage, patients were divided in two categories: 33 patients were T1–2, 59 were T3–4 and one patient had unknown T stage. As for lymph node status, 38 patients had positive lymph node metastasis, and 55 had negative status (N0). The TNM stage distribution was as follows: there were stage I–II 26, stage III–IV 66, and unknown 1. Thirteen patients were classified as NET and 80 were NEC (Table 1). For S + RT group, 25 of the 34 patients underwent microscopically margin-negative resection (R0) and the others suffered positive surgical margins. For the 19 patients in S + Sa group, 18 received primary R0 resection. After the first-time recurrence, 6 received salvage surgery, 2 underwent salvage surgery plus post-operative radiotherapy, 3 received salvage radiotherapy, and 2 received palliative radiotherapy or chemotherapy.

**Table 1:** Patients’ characters

Factors	C + RT (*n* = 40, %)	S + RT (*n* = 34, %)	S + Sa (*n* = 19, %)	*χ*^2^	*p*
Age					
≤ 50	21 (52.5)	16 (47.1)	3 (15.8)	7.441	**0.024**
> 50	19 (47.5)	18 (52.9)	16 (84.2)		
Gender					
Male	31 (77.5)	24 (70.6)	16 (84.2)	1.304	0.521
Female	9 (22.5)	10 (29.4)	3 (15.8)		
Primary tumor site					
Nasal cavity and paranasal sinus	21 (52.5)	20 (58.8)	3 (15.8)	26.582	**0.000**
Larynx	4 (10.0)	6 (17.6)	13 (68.4)		
Others	15 (37.5)	8 (23.5)	3 (15.8)		
T stage					
T1–2	7 (17.5)	15 (44.1)	11 (57.9)	10.457	**0.005**
T3–4	32 (80.0)	19 (55.9)	8 (42.1)		
Unknown	1 (2.5)	–	–		
N status					
Negative	20 (50.0)	22 (64.7)	13 (68.4)	2.496	0.287
Positive	20 (50.0)	12 (35.3)	6 (31.6)		
Stage (AJCC/UICC)					
I–II	4 (10.0)	12 (35.3)	10 (52.6)	12.631	**0.002**
III–IV	35 (87.5)	22 (64.7)	9 (47.4)		
Differentiation					
NET	2 (5.0)	4 (11.8)	7 (36.8)	11.080	**0.004**
NEC	38 (95.0)	30 (88.2)	12 (63.2)		

*p* values in bold incidate the existence of statistically significant difference

*C* chemotherapy, *RT* radiotherapy, *S* surgery, *Sa* salvage, *T* tumor, *N* lymph node, *n* number, *NET* neuroendocrine tumor, *NEC* neuroendocrine carcinoma

### Treatment outcomes

**Fig. 1:**
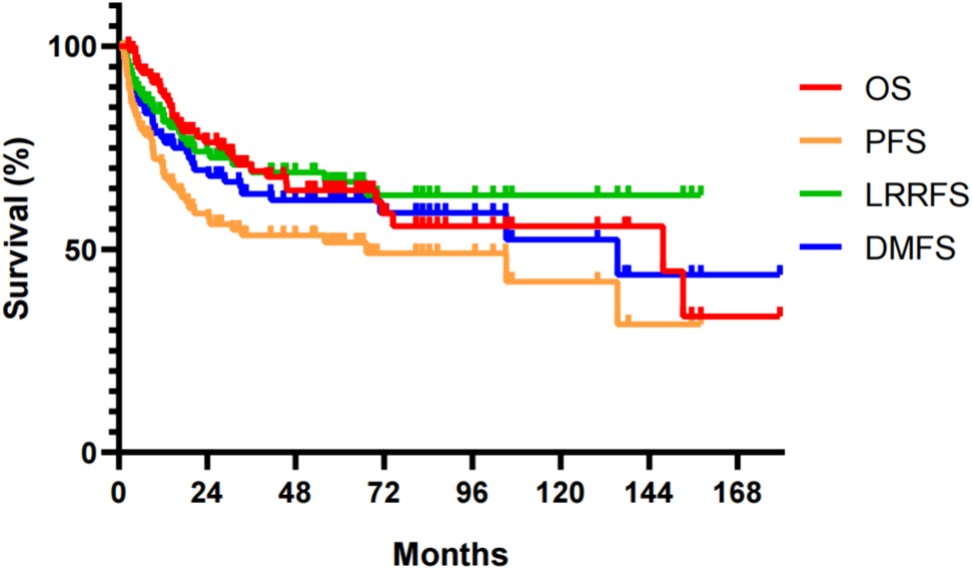
Survival curves (OS, PFS, LRRFS and DMFS) for HNNENs

With the median follow-up time of 65 (range from 3 to 180) months, 5-year OS, PFS, LRRFS, and DMFS of HNNENs were 64.5%, 51.6%, 66.6%, 62.1%, respectively, as shown in Fig. 1.

**Fig. 2:**
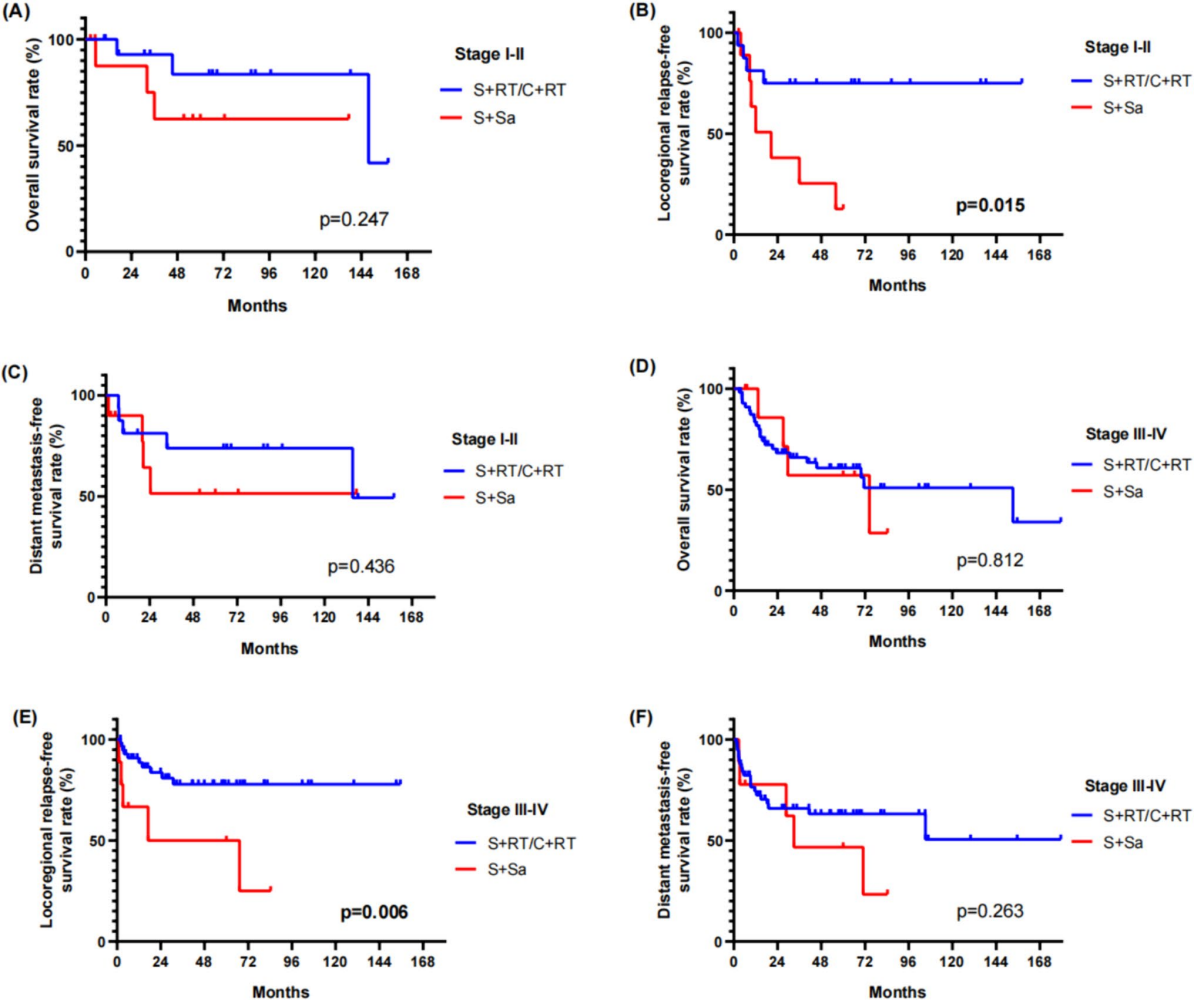
Survivals among the patients received RT vs non-RT. **A** OS; **B** LRRFS; **C** DMFS of stage I–II patients; **D** OS; **E** LRRFS; **F** DMFS of stage III–IV patients

According to TNM stage and treatment models, further analysis was performed. For patients with stage I–II, 5-year OS, LRRFS, and DMFS for those who underwent radiotherapy (including C + RT and S + RT groups) were 83.6%, 75.0%, and 73.9%, and for those who did not receive planned radiotherapy (S + Sa group), 5-year OS, LRRFS, and DMFS were 62.5%, 12.7%, and 51.4%, respectively. For patients with stage III–IV, 5-year OS, LRRFS, and DMFS for radiotherapy group (including C + RT and S + RT groups) were 60.7%, 77.8%, and 63.2%, and for S + Sa group, 5-year OS, LRRFS, and DMFS were 57.1%, 50.0%, and 46.7%, respectively. As shown in Fig. 2, the combination of RT with chemotherapy or surgery elicited superior local control whether the tumor stage was early (*p* = 0.015) or late (*p* = 0.006), but did not significantly improve the overall survival or distant metastasis-free survival.

**Fig. 3:**
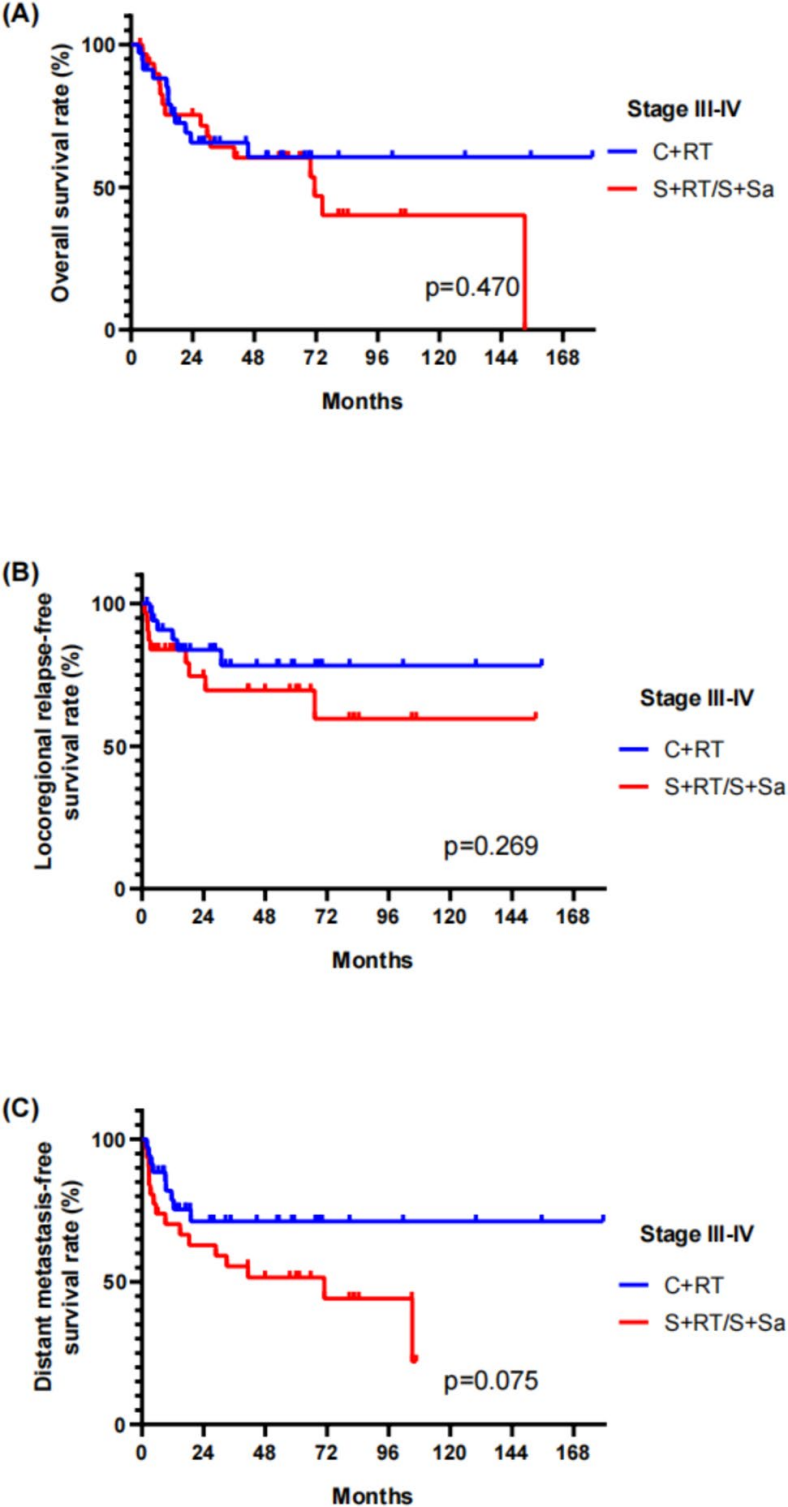
Survivals among the patients received systemic chemotherapy vs non-chemotherapy. **A** OS; **B** LRRFS; **C** DMFS of stage III–IV patients

To explore the role of systemic therapy, we performed further analysis on patients with stage III–IV. The individuals were re-grouped according to the application of systemic therapy, including adjuvant or neoadjuvant chemotherapy. 5-year OS, LRRFS, and DMFS for patients who received systemic therapy (C + RT group) were 60.6%, 78.2%, and 71.2%, and for the non-systemic therapy group (S + RT or S + Sa), 5-year OS, LRRFS, and DMFS were 60.3%, 69.6%, and 51.5%, respectively. Although there was no statistical difference (*p* = 0.075), patients who received systemic therapy had better DMFS than those did not receive systemic therapy (Fig. 3).

### Failure patterns

**Fig. 4:**
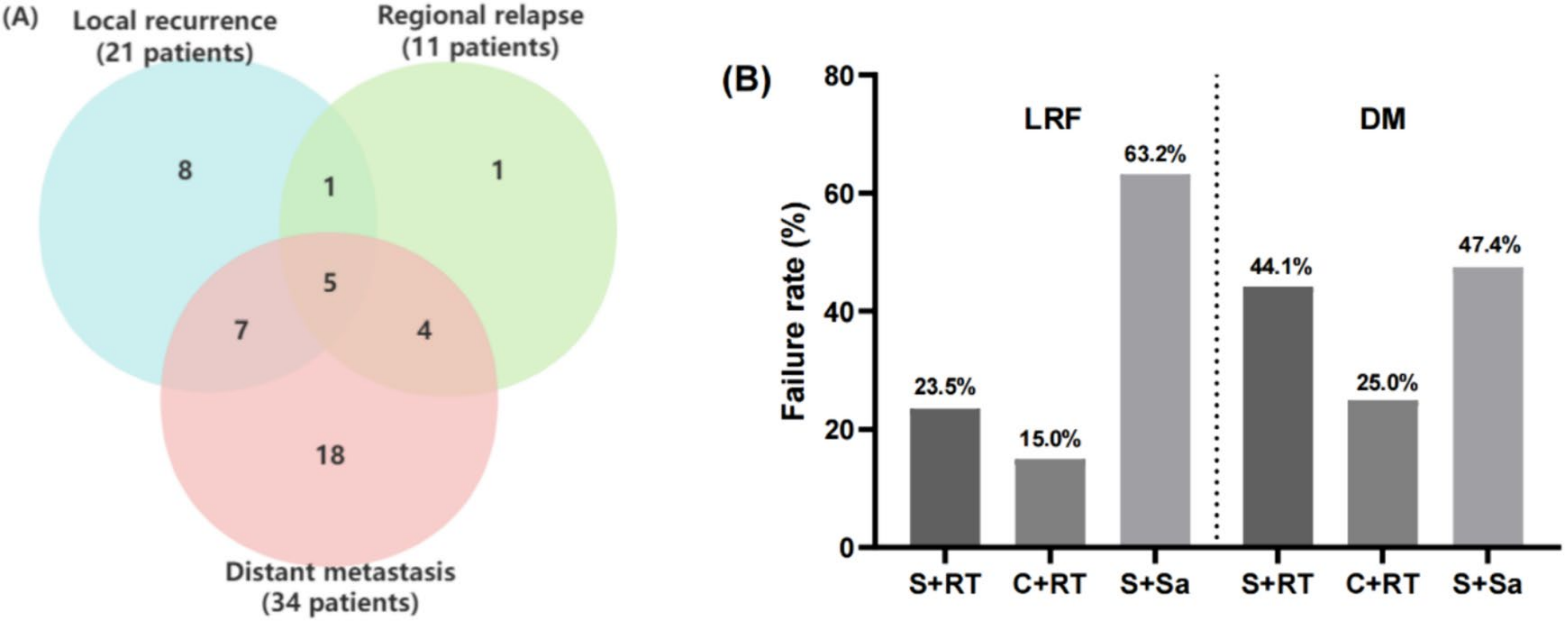
Failure patterns **A** of the HNNENs; **B** among the various treatment modalities

Totally, 44 of the 93 patients experienced treatment failure, and the failure patterns are shown in Fig. 4A. Local recurrences were found in 21 patients and regional failure occurred in 11 patients. The most common sites of nodal failure were level II, followed by level III. 34 patients developed distant metastasis, the common sites were liver, lung, bone, and central nervous system. In addition, we analyzed the patterns of treatment failure in patients who experienced different treatment options (Fig. 4B). Patients who received planned S + RT or C + RT had obviously lower loco-regional control failure (LRF) than S + Sa group (23.5% and 15.0% vs 63.2%, *p* < 0.001). The difference in distant metastasis (DM) between the three groups did not reach statistical significance (*p* = 0.129).

### Prognostic analysis

The results of univariate analyses showed that differentiation degree had significant effect on OS and DMFS rather than LRRFS. Treatment regimen was a significant prognostic factor affecting LRRFS (Table 2). The multivariate analyses (Table 3) indicated that there was no independent predictor of overall survival. Among the various treatment modalities, S + Sa was used as a reference, and both the C + RT group and the S + RT group exhibited a significant improvement in LRRFS (C + RT group: HR, 0.152, range 0.047–0.492, *p* = 0.002 and S + RT group: HR, 0.227, range 0.078–0.656, *p* = 0.006). In addition, degree of differentiation and treatment including chemotherapy or not were independent predictors of DMFS.

**Table 2:** Univariate analysis

Factors	patients	5-year OS (%)	*p*	5-year LRRFS (%)	*p*	5-year DMFS (%)	*p*
Age							
≤ 50	40	73.0	0.097	71.3	0.258	69.1	0.262
> 50	53	57.0		63.5		54.9	
Gender							
Male	71	60.2	0.121	66.9	0.689	56.1	0.077
Female	22	78.3		65.4		80.5	
Primary tumor site							
Nasal cavity and paranasal sinus	45	61.2	0.921	64.5	0.228	64.2	0.165
Larynx	23	64.2		54.1		46.5	
Others	25	74.5		83.2		74.3	
T stage							
T1–2	33	66.8	0.388	53.5	0.117	60.3	0.890
T3–4	59	63.0		74.5		62.7	
Unknown	1	–		–		–	
N status							
Negative	55	66.8	0.322	59.6	0.265	63.7	0.223
Positive	38	60.9		81.8		59.7	
Stage							
I–II	26	75.4	0.098	51.7	0.155	65.0	0.448
III–IV	66	60.3		74.0		60.6	
Unknown	1	–		–			
Degree of differentiation							
NET	13	100	**0.008**	55.6	0.667	90.9	**0.022**
NEC	80	58.2		69.6		57.1	
Treatment							
C + RT	40	61.3	0.922	81.5	**0.000**	71.1	0.245
S + RT	34	70.5		73.2		59.9	
S + Sa	19	60.5		28.5		48.8	

*p* values in bold incidate the existence of statistically significant difference

*OS* overall survival rate, *LRRFS* locoregional relapse-free survival free rate, *DMFS* distant metastasis-free survival rate, *C* chemotherapy, *RT* radiotherapy, *S* surgery, *Sa* salvage, *T* tumor, *N* lymph node, *NET* neuroendocrine tumor, *NEC* neuroendocrine carcinoma

**Table 3:** Results of COX multivariate analysis

Factors	OS		LRRFS		DMFS	
	HR (95% CI)	*p*	HR (95% CI)	*p*	HR (95% CI)	*p*
Age						
≤ 50/> 50	0.479 (0.219–1.050)	0.066	0.954 (0.380–2.394)	0.920	0.787 (0.358–1.730)	0.550
Gender						
M/F	1.213 (0.451–3.260)	0.702	1.147 (0.411–3.197)	0.793	1.553 (0.570–4.227)	0.389
Stage						
I–II/III–IV	0.510 (0.202–1.292)	0.156	0.831 (0.354–1.950)	0.671	0.763 (0.336–1.733)	0.518
Degree of differentiation						
NET/NEC	< 0.001 (–)	0.964	0.526 (0.172–1.605)	0.259	0.104 (0.013–0.812)	**0.031**
Treatment						
S + Sa	1		1		1	
C + RT	0.607 (0.223–1.654)	0.329	0.152 (0.047–0.492)	**0.002**	0.305 (0.109–0.854)	**0.024**
S + RT	0.742 (0.276–1.992)	0.553	0.227 (0.078–0.656)	**0.006**	0.608 (0.236–1.566)	0.302

*p* values in bold incidate the existence of statistically significant difference

*OS* overall survival rate, *LRRFS* locoregional relapse-free survival free rate, *DMFS* distant metastasis-free survival rate, *M* male, *F* female, *HR* hazard ratio, *CI* confidence interval, *C* chemotherapy, *RT* radiotherapy, *S* surgery, *Sa* salvage, *Sa* neuroendocrine tumor, *NEC* neuroendocrine carcinoma

## Discussion

HNNENs remain a rare disease and relevant reports are primarily case reports. The present study included 93 cases of HNNENs, which constitutes one of the largest single-institution cohorts of HNNENs patients.

Most epidemiological and clinical data in our study are comparable to those reported in previous studies. As described in previous studies, males have been more likely to suffer from this disease than females, the male–female ratio ranged from 1.5:1 to 8:1 (Bal et al. 2022; Ghosh et al. 2015; Issa et al. 2021; Kao et al. 2012; Mitchell et al. 2021; Pointer et al. 2017; Yu et al. 2020). The ratio in our study is 3.2:1, which is consistent with previous reports. The median age at diagnosis ranges from 57 to 64 years (Bal et al. 2022; Ghosh et al. 2015; Issa et al. 2021; Kao et al. 2012; Mitchell et al. 2021; Pointer et al. 2017; Yu et al. 2020). In our study, most of the HNNENs were located in the nasal cavity and paranasal sinus (45/95), followed by the larynx (23/95). This result is consistent with a previous study based on the Surveillance, Epidemiology, and End Results (SEER) database from 1973 to 2012 (Kuan et al. 2017). Meanwhile, there are some contrasts as well. Another study based on the SEER database analyzed a total of 789 cases of small cell carcinoma of the head and neck from 1973 to 2016, reporting that the highest incidence sites were salivary glands (over 25.0%) and nasal cavity and paranasal sinus (18.8%) (Yu et al. 2020). One study on the NCDB database analyzed a total of 1042 cases of small cell carcinoma of the head and neck from 2004 to 2012, the high-incidence areas were as follows: larynx (34.9%), nasal cavity and paranasal sinus (30.0%), and oropharynx (12.3%) (Pointer et al. 2017). We believe that differences in race may also contribute to this result. The symptoms of HNNENs are usually non-specific and mostly depend on the site of the primary tumor. Thus, the final diagnosis depends on pathological characters and genomic features (Ohmoto et al. 2021; Perez-Ordoñez 2018).

Survival rate of HNNENs reported in previous literature has great differences and is closely related to the degree of differentiation. Despite that in the latest WHO 2022 classification, the terms “typical carcinoid” (well-differentiated) and “atypical carcinoid” (moderately differentiated) were de-emphasized and used as synonyms only, and they were the preferred terminologies in previous researches (Mete and Wenig 2022; Perez-Ordoñez 2018). Patients with typical carcinoid of head and neck usually had very good prognosis, and the 5-year disease-specific survival rates of currently reported cases are almost 100% in previous reports (Kao et al. 2012; van der Laan et al. 2015). For atypical carcinoid, the 5-year survival rates reported by previous studies ranged from 46 to 83.3% (Ferlito et al. 2006; Kao et al. 2012; van der Laan et al. 2015). Moreover, HNNECs are more aggressive and had worse prognosis, with higher local recurrence rate and distant metastasis rate, reducing the 5-year overall survival rate to the range of 5–34% (Ghosh et al. 2015; Kao et al. 2012; Lin et al. 2019; van der Laan et al. 2015; Yu et al. 2020). We observed that 5-year OS in our study was higher than historical reports, which could be due to differences in treatment strategies and radiation dose or the small number of patients included in many of the previous studies. The median CTV dose in our study was 60.06 Gy, which is higher than previous studies. And as reported, the radiation dose more than 40Gy was associated with improved OS (Lin et al. 2019). In addition, no patients had distant metastases at the time of diagnosis, which would also contribute to the higher survival rate. A study based on SEER database included 789 primary cases and reported 5-year OS rate was 26.2% (Yu et al. 2020). It was noted that almost 20% of the patients included in this study had distant metastases at the time of diagnosis, which may be the main reason of the lower survival rates.

Treatment modalities for HNNENs included surgery, radiotherapy, chemotherapy, or the combination of two or more treatment types. Despite no consensus on the optimal treatment currently, it is widely recognized that treatment options largely depend on degree of differentiation and clinical stage. Typical carcinoid of the head and neck can be cured with local excision or radiotherapy (Kao et al. 2012; van der Laan et al. 2015), while the best treatment for atypical carcinoid and HNNECs remained unclear (Mitchell et al. 2021; van der Laan et al. 2015). Since surgery and radiotherapy are both local treatments, some scholars have compared them and emphasized the importance of surgery. But in the study, patients underwent surgery and adjuvant radiotherapy were also included in the surgery group (Patel et al. 2015), which may cause bias. In our study, when compared to patient-treated regimen with or without RT, RT significantly improved LRRFS both in early and late stages despite no improvement in OS and DMFS. Thus, we argued that RT plays an important role in local control of HNNENs and surgery alone cannot achieve satisfactory results, no matter early or late stage. In further analysis, systemic chemotherapy improved DMFS though the result did not reach statistically significance. Based on the above results, we believed that systemic treatment is still indispensable. Similarly, a meta-analysis (van der Laan et al. 2015) suggested that atypical carcinoid needs to extend the follow-up period to 10 years to observe late recurrences to determine optimal treatment, while the small or large cell neuroendocrine carcinoma seems to benefit more from a combination of radiotherapy and chemotherapy. Consistently, another study based on SEER database investigated the 5-year disease-specific survival (DSS) of the various treatment groups for small cell HNNECs, the 5-year DSS was 26% for patients receiving surgery alone, 32% for those treated by surgery and radiotherapy, and 40% for those undergoing surgery, radiotherapy and chemotherapy (Yu et al. 2020). But there was no statistically significant difference (*p* = 0.21) for the different treatment modalities (Yu et al. 2020).

Previous studies have reported that distant metastasis was the main failure pattern, and liver and lung were the most common sites of metastasis (Ferlito et al. 2006; Thompson et al. 2016). Our study supported these results. In addition, we also observed relatively high rate of bone and central nervous system metastases, which might suggest a role for chemotherapy. Moreover, we found that patients who received RT had obviously lower LRF than S + Sa group. Systemic chemotherapy was related with lower DM though there was no statistical significance. This result may be related to the small sample size.

The prognosis of HNNENs is related to many factors, such as patient characters and tumor variables. Independent prognostic indicator included N stage, M stage, AJCC stage, and chemotherapy had been reported in previous studies (Yu et al. 2020). Some researchers reported that primary tumor sites had important impact on prognosis, for example, it was reported that sinonasal primaries appear to have better survival compared to other primary sites within head and neck (Kuan et al. 2017). Tom et al.’s meta-analysis (van der Laan et al. 2016) concluded that differentiation grade was one of the most important predictors of survival in sinonasal neuroendocrine carcinoma and was associated with choice of treatment modality. They also found that tumor staging appeared limited in value in predicting survival (van der Laan et al. 2016). Results of univariate analysis in our study showed a degree of differentiation which had an effect on OS and DMFS rather than LRRFS. The multivariate analysis revealed that S + RT or C + RT group had obviously better LRRFS while C + RT group had better DMFS. Thus, we recommend comprehensive treatment for HNNENs and individualized treatment plans for different patients.

Some limitations were noted in our study. First, it was a retrospective study which was based on data from a single hospital over a long period of time. Therefore, we cannot rule out some degree of selection bias. Second, although this study included, as we known, the largest sample size in single institution, we were still unable to conduct more detailed subgroup analysis such as different primary tumor sites or various resection margin status. We expected more studies with bigger sample size and more data in future.

## Conclusion

Radiotherapy improved local–regional control and played an important role in the management of HNNENs. The optimal treatment regimen for HNNENs remains the combination of local and systemic treatments.

## Data Availability

The data underlying this article will be shared on reasonable request to the corresponding author.
